# The Prevalence and Associated Factors of Hypertension among HIV Patients

**DOI:** 10.1155/2021/5544916

**Published:** 2021-04-15

**Authors:** Grace Wambura Mbuthia, Karani Magutah, Stephen T. McGarvey

**Affiliations:** ^1^College of Health Sciences, Jomo Kenyatta University of Agriculture and Technology, P.O. Box 62000- 0200, Nairobi, Kenya; ^2^School of Medicine, College of Health Sciences, Moi University, P.O. Box 4606 Nairobi, Kenya; ^3^International Health Institute, Department of Epidemiology, School of Public Health, and Department of Anthropology, Brown University, Providence, Providence, RI 02912, USA

## Abstract

**Background:**

The dual burden of cardiovascular diseases and Human Immunodeficiency Virus (HIV) in sub-Saharan Africa is of public health concern. Persons living with HIV are 1.5–2 times more likely to develop CVD risk factors compared to the noninfected individuals. Hypertension is a major risk factor leading to the rising CVD epidemic in SSA. However, the burden of hypertension among HIV patients in Kenya is not well documented.

**Objective:**

This study determined the prevalence and the associated factors of hypertension among HIV patients receiving regular care at Thika Level 5 Hospital Comprehensive Care Clinic (CCC), within metropolitan Nairobi, Kenya.

**Methods:**

The current cross-sectional study involved review of patients' records/charts. Charts for adult patients seen in the last 6 months at Thika Level 5 Hospital CCC were included in the study. Hypertension was defined as systolic blood pressure ≥140 mmHg and/or diastolic blood pressure ≥90 mmHg on two different readings one month apart, while overweight/obesity was defined as body mass index (BMI) ≥ 25 kg/m^2^.

**Results:**

In a sample of 939 HIV patients, the majority, 68.8% (646), were female. The patients' ages ranged from 18 to 84 years with a median age of 44 (IQR 37–51) years. The mean BMI was higher for females (25.8 kg/m^2^) compared to that of males (23.1 kg/m^2^). However, the prevalence of hypertension was higher among males (25.3%) compared to females (16.9%). Age >40 years (AOR = 2.80, *p* ≤ 0.001), male sex (AOR = 2.10; *p*=0.04), history of alcohol consumption (AOR = 2.56, *p* ≤ 0.001), and being overweight/obese (AOR = 2.77 *p* ≤ 0.001) were significantly associated with hypertension. The antiretroviral (ARV) regimen and, additionally, the duration of antiretroviral therapy had no association with being hypertensive.

**Conclusion:**

The prevalence of hypertension is high among HIV patients. Traditional cardiovascular risk factors were associated with hypertension, but no association was observed with ART regime or duration of ARV use. There is a need to integrate hypertension management into regular HIV care.

## 1. Background Information

The dual burden of cardiovascular diseases (CVDs) and human immunodeficiency virus (HIV) in sub-Saharan Africa (SSA) is of public health concern. Of the 38 million people currently living with HIV worldwide [1], it is estimated that nearly 70% live in SSA [2]. At this same time, there is a growing burden of noncommunicable diseases (NCDs) in the SSA's general population which poses a big challenge to the weak health systems in these resource limited settings [3]. Noncommunicable diseases (NCDs) account for more than 50% of total hospital admissions and over 55% of hospital deaths in Kenya. The 2015 Kenya STEPwise survey for NCD risk factors showed that the prevalence of hypertension and overweight/obesity is high among adults aged 18–69 years at 24% and 28%, respectively [4].

With the demographic and epidemiological transition from infectious diseases to NCDs in SSA, there is a growing burden of CVD due to, among other factors, rapid urbanization and economic changes and concomitant nutrition transitions [5]. Moreover, HIV-infected individuals have an increased risk of CVD risk factors and cardiovascular morbidity and mortality outcomes compared to noninfected individuals [6]. The increased risk has been linked to the traditional CVD risk factors, HIV infection itself, antiretroviral regimen, and the immunological dynamics that originate from the infection [6, 7]. A systematic review and meta-analysis among HIV-infected populations in SSA countries indicated a strong association between the use of antiretroviral therapy (ART) and mortality due to CVD [8]. Similarly, meta-analysis has shown HIV infection as a risk factor for vascular diseases with variation in different regions [9]. This has been attributed to the aging population of persons living with HIV, ART, HIV-related inflammation, and traditional lifestyle risk factors such as smoking, alcohol intake, and inactivity [6, 10, 11].

Studies on the association of cardiovascular risk factors and HIV are mainly from the developed countries [12–16], yet SSA bears 67% of the global burden of HIV [2]. Analyzing the distribution of cardiovascular risk factors among persons living with HIV in SSA is important due to the known cardiovascular effects of HIV and the high prevalence of HIV.

As a result of the success and scale-up of ART over the past fifteen years, HIV has become a chronic disease with increased life expectancy and aging among patients in SSA [17, 18]. The use of highly active antiretroviral therapy (HAART) has been linked to hyperglycemia, dyslipidemia, and increased risk of CVD in HIV-infected patients [19]. Similarly, newer first- and second-line regimens containing dolutegravir replacing older antiretroviral therapies with less efficacy and greater toxicity has been associated with hyperglycemia [20, 21]. However, the burden of CVD risk factors among HIV patients is not well documented in Kenya [22]. Previous studies on cardiovascular risk and HIV comorbidity, mainly from Western Kenya, have shown a high prevalence of hypertension and obesity among HIV patients [23, 24]. Similarly, a clinic based study in Western Kenya showed an elevated prevalence of undiagnosed cardiovascular risk factors such as hypertension (13.3%) and raised total cholesterol levels (14%) among HIV patients [25]. There is a need for further studies to demonstrate the magnitude of the problem in Kenya and inform interventions geared to the prevention of CVD outcomes among HIV patients. Additionally, there is a need for further studies to examine the effect of increasing use of HAART on cardiovascular risk factors following the adoption of the “test and treat” World Health Organization (WHO) HIV treatment guidelines in the year 2016 [26, 27]. The purpose of this study was to determine the prevalence of hypertension and the associated factors among a predominantly urban population of adult HIV patients residing within the Nairobi metropolitan region.

## 2. Methods

### 2.1. Study Setting

The study was conducted at Thika Level 5 Hospital (TL5H) HIV Comprehensive Care Clinic (CCC). The hospital is a county referral hospital serving wider Kiambu and neighboring counties and is located in Thika town, 42 kilometers North East of Kenya's biggest city, Nairobi. Thika is an industrial city center with a population of 279,429. The CCC had a total of 4,500 active patients with an average monthly enrolment of 42 patients in the year 2016 [28]. Currently, the clinic has approximately 10,000 active patients (unpublished clinic report). The HIV prevalence in Kiambu county is 4%, with a total of 34,417 adults on ART treatment [29].

### 2.2. Study Design

This was a cross-sectional hospital based survey of patients' charts and in-depth interviews at the TL5H CCC.

### 2.3. Study Population

The study involved adult HIV-infected patients receiving HIV care at TL5H.

### 2.4. Inclusion Criteria and Exclusion Criteria

Charts for adult HIV-infected patients attending TL5H CCC over a six-month period, March to September 2020, were chosen and abstracted for the study. Charts for pregnant HIV patients and those with missing details on key variables such as recorded blood pressure and ART regimen were excluded from the data.

### 2.5. Sample Size Calculation

The minimum required sample size was estimated using Cochran's formula (1977) with the following assumptions: 24% prevalence of hypertension (the prevalence of hypertension among adults aged 18–69 years in Kenya [4]), 5% precision, and a 95% confidence interval as follows:

(1) $$\begin{array}{c} n_{0}=\frac{Z^{2}pq}{e^{2}}\\ =\frac{1.96^{2}\times 0.23\times 0.77}{{\left(0.05\right)}^{2}}\\ =272, \end{array}$$

where *e* is the desired level of precision (i.e. the margin of error), *p* is the (estimated) proportion of the population which has the attribute in question, and *q* is 1−*p*.

The minimum required sample was 272 medical records.

However, to increase the precision of the study, we targeted 10% of all the registered patients in the clinic and reviewed a total of 1000 records. Only 939 were complete for analysis and were included in this study.

### 2.6. Sampling Technique

Systematic random sampling was used to select files of HIV-infected patients attending routine clinic visits at TL5H. Charts of patients seen in the six-month study period were included in the study. The CCC receives 75 patients on average daily, translating to about 9000 patients in 6 months. Since all patient charts receive numbers that are serially allocated as patients are attended, we arranged these serial numbers in a database file and selected every 9th chart until the 1000 charts were selected from the registry for medical records abstraction.

#### 2.6.1. Data Collection Tools

Data collection was done using a standardized electronic extraction form. The variables included in the tool were sociodemographic characteristics, biological risk factors (blood pressure, weight, waist circumference, body mass index (BMI), lipids profile, and blood glucose), lifestyle risk factors such as the history of smoking and alcohol intake, and ongoing ART therapy. The documented blood pressure (BP) was measured routinely using a hospital-grade Omron M3® (Omron, Netherlands) digital automatic blood pressure machine.

#### 2.6.2. Data Collection Procedure

Data collection involved the extraction of data from the patients' charts using a standardized data collection tool by trained research assistants. Available data were extracted to an electronic data extraction tool and later transferred into Stata software (College Station, TX: StataCorp LP) for data cleaning and analysis. The clinical data for each patient were drawn from the most recent available patient encounter for each variable separately.

### 2.7. Data Analysis

The *Stata* version 15 (College Station, TX: StataCorp LP) computer statistical package was used for data analysis. Descriptive statistics were used to define the participants' characteristics. Categorical data were analyzed and reported in frequency and percentages. Continuous data were analyzed using measures of central tendencies. Blood pressure was categorized as either normal (systolic <130 and/or diastolic < 85), prehypertensive (systolic 130–139 mmHg and/or diastolic 85–89 mmHg), or hypertensive (systolic ≥ 140 and/or diastolic ≥ 90 mmHg) according to the Kenyan national guidelines for cardiovascular diseases management [30]. Obesity was determined by the WHO BMI categorization: underweight (BMI <18.5), normal weight (BMI 18.5–24.9), and overweight/obesity (BMI ≥ 25).

Bivariate analysis of risk factors was done using chi-square test as a measure of association and the statistical significance was considered at *p* < 0.05. Logistic regression was used to analyze the sociodemographic and clinical factors associated with hypertension.

#### 2.7.1. Operational Definition

Hypertension is defined as systolic blood pressure (SBP) ≥ 140 mmHg and/or diastolic blood pressure (DBP) ≥ 90 mmHg on 2 different readings at least 1 month apart.

At the time of this study, the management for hypertension and other noncommunicable diseases at TL5H had not been integrated into the regular HIV care and data on hypertension treatment was missing in the patient's charts.

#### 2.7.2. Study Limitations

The limitations of the present study relate to the cross-sectional design that relied on secondary data from patients' charts. As such, the results presented imply associations only, and causality cannot be drawn from the findings. In addition, information on hypertension treatment and important risk factors such as physical activity, diet, and salt intake was not collected due to the incomplete medical data.

### 2.8. Ethical Considerations

The study was approved by Moi Teaching and Referral Hospital/Moi University research ethics committee (approval number FAN: 0003637). Similarly, approval to use the CCC medical data was sought and granted by the TL5H administration. Confidentiality and anonymity of patients were guaranteed by excluding unique identifiers from the data collected from the patients' charts. Informed consent was not sought from the patients as the study utilized secondary data.

## 3. Results

### 3.1. Sociodemographic and Clinical Characteristics of the Respondents

The majority of the patients (68.8%) were women. The respondents' age ranged from 18 to 84 years, with mean age 44 (10.7) years and median age of 44 (IQR 37–51) years. The mean SBP and DBP for the men (127.9 ± 17.9 mmHg, 75.5 ± 11.7 mmHg) were slightly higher than that of the women (121.3 ± 17.8 mmHg, 73.4 ± 10.1 mmHg). The prevalence of hypertension was higher among men (25.3%) compared to women (16.9%). Similarly, a higher proportion of men (15.0%) were prehypertensive compared to women (13.5%).

However, the mean BMI was higher for the women (25.8 ± 5.1 kg/m^2^) compared to that of the males (23.1 ± 4.2 kg/m^2^). Using the WHO BMI classification, the prevalence of overweight/obesity was also higher among females (53.7%) compared to that for males (28.3%). A small proportion of the patients, 7.1% (67), were underweight.

All the patients were on ART therapy with the majority (88%) having been on treatment for more than 2 years. A majority, 65% (614), was on the standard first-line HIV treatment regime in Kenya, which consist of two nucleoside reverse transcriptase inhibitors (NRTI), mainly tenofovir disoproxil fumarate (TDF) or azidovudine (AZT) or stavudine (D4T), lamivudine (3TC) and one nonnucleoside reverse transcriptase inhibitors efavirenz (EFV) or niverapine (NVP) while a minority, 5% (47), had protease inhibitor (PI) containing regimen. Approximately one-third (30%) of the patients had a regimen containing a combination of two nucleoside reverse transcriptase inhibitors and one integrase strand transfer inhibitor (INSTI) dolutegravir (DTG). Dolutegravir containing regimen was more common among men 55.5% (163) compared to 18% (115) for the women.

Only a small proportion of the patients, 6% (54), had a history of smoking, and 11% (95) of the patients had a history of alcohol consumption. Other characteristics are as shown in Table 1.

#### 3.1.1. Factors Associated with Hypertension among HIV Patients

Table 2 shows that sex (*χ*^2^ = 11.4 *p*=0.001), age (*χ*2 = 62.6 *p* ≤ 0.001), marital status (*χ*2 = 13.6 *p*=0.03), BMI (*χ*2 = 33.9 *p* ≤ 0.001), and ARV regimen (*χ*2 = 8.2 *p*=0.02) of the patient and history of alcohol consumption were significantly associated with hypertension in unadjusted bivariate analyses. The duration of ART therapy and the history of smoking were not associated with hypertension.

After adjusting for confounding factors, being male, older than 40 years of age, overweight, obese, and having history of alcohol consumption were independently and significantly associated with hypertension (Table 3).

The male patients were 2 times more likely to have hypertension compared to the females. Similarly, patients aged 41–50 years, 51–60 years, and those aged ≥50 years had a 3-fold, 5-fold, and 6-fold greater odds of having hypertension, respectively, compared to patients aged 18–40 years. Overweight and obesity were associated with 2-fold and 3-fold greater odds of having hypertension, respectively, compared to patients with normal BMI. Similarly, patients who consumed alcohol were 3 times more likely to be hypertensive compared to those who did not.

History of smoking, ART regimen, and duration of ART therapy were not significantly associated with being hypertensive (Table 3).

## 4. Discussion

This cross-sectional study of HIV + patients' charts from metropolitan Nairobi, Kenya, is among the few aimed to assess the prevalence and factors associated with hypertension following the implementation of the WHO “test and treat” HIV guidelines [27] in an urban sub-Saharan African setting. Key findings were a high prevalence of hypertension of 25% and 16% among male and female HIV patients, respectively, high prevalence of overweight/obesity of 26.6% among males and 50.4% among female HIV patients, and the association of well-known cardiovascular risk factors with hypertension. There were no associations of hypertension with the type of ART regimen or duration of ART use.

As countries work towards achieving the United Nations HIV goals of 90–90–90 (90% of people with HIV diagnosed, 90% of them on ART, and 90% of them having viral suppression by the year 2020), there has been a reduction in HIV-related diseases while NCDs have become prevalent among persons infected with HIV [31]. The emerging burden of NCDs among HIV patients is a result of numerous factors, among them the now increased life expectancy and aging among HIV patients and the rise of the traditional lifestyle related CVD risk factors such as diet, obesity, smoking, and alcohol intake in the general population in the context of urbanization, and economic developments [17, 18, 32]. This double burden of disease represents a major strain for still underresourced health services in low and middle income countries.

The current study shows a high prevalence of hypertension among HIV patients, much higher than the prevalence shown in previous studies done in Kenya among HIV patients [22, 23, 25]. In a nationally representative retrospective medical chart review of 3170 HIV-infected adults enrolled in HIV care in Kenya from 2003 to 2013, [22] found a prevalence of 11.5% of NCDs with elevated blood pressure being the most common NCD among people living with HIV. Similarly, a retrospective analysis of 12,194 electronic medical records of adult HIV patients enrolled in a treatment program in Western Kenya between 2006 and 2009 found a prevalence of hypertension of 11.2% and 7.4%, among men and women, respectively [23]. In yet another study, [25] found a prevalence of 13% for hypertension among adult HIV patients enrolled in care between 2013 and 2015 at Ukwala subcounty hospital in Western Kenya. These studies show a growing trend of the burden of this cardiovascular risk among HIV patients in Kenya. However, the prevalence is lower than the prevalence of 29.5% documented in a study done in Ethiopia among HIV patients on HAART [33]. The high prevalence of hypertension suggests that a substantial number of cardiovascular diseases could be prevented by improving blood pressure control.

The increased risk of hypertension observed among HIV-infected patients with standard cardiovascular risk factors such as older age, male gender, overweight/obesity, and alcohol consumption in this study is consistent with previous studies conducted among HIV-infected patients [23, 34, 35]. However, in the current study, we did not find any association between hypertension and history of smoking among HIV + individuals. This could be attributed to the fact that there was low power to detect such associations considering the small number and low percentage of patients, 5.8%, almost all men (see Table 1), who had a history of smoking recorded in the charts. Further research is needed at the hospital to determine how tobacco smoking information is regularly collected among HIV + patients.

The current study did not find any association between the type of ARV drugs used or the duration of ART therapy and hypertension. This is in agreement with a systematic review and meta-analysis on the association between ART and cardiovascular disease risk factors in SSA that found no direct association between hypertension and ART use [8]. However, the current findings are contrary to other studies that have shown an association of the duration of ART use and hypertension [33, 36, 37]. According to [8], the observed association may not be directly due to hypertension but could be due to dyslipidemia associated with ART use. At the time of this study, Kenya was implementing the WHO “test and treat” guidelines which recommend ART for all PLHIV regardless of WHO stage, CD4 count, age, pregnancy status, or comorbidities, and therefore, all the HIV patients were on HAART. The majority of the patients were on the treatment regime in Kenya containing two NRTI and one NNRTI, while a third of the patients were on DTG containing regimen. Dolutegravir, an integrase strand transfer inhibitor (INSTI) containing regimen, has become a preferred first-line HIV treatment because of its efficacy, tolerability, limited drug-drug interactions, and a high barrier to resistance [38, 39]. In this study, a majority of the patients on DTG containing regimen were males owing to the fact that in Kenya, DTG is not currently recommended for women of childbearing potential [40, 41] due to its questionable safety during pregnancy [42, 43] and its associated adverse weight gain in pregnancy [44]. Further studies of DTG and hypertension and other CVD risk factors are needed as DTG becomes more widely used.

In this study, we were not able to establish whether the patients with hypertension were on hypertension treatment and regular follow-up given the care of NCDs was offered in the medical outpatient clinic with the rest of the patients separate from the HIV CCC. This also indicates a lack of awareness on the importance of monitoring and management of CVD risk factors among this group of HIV + patients. Hypertension and other NCDs have similarities with HIV, such as being a chronic disease that require regular follow-up and optimal treatment adherence and the importance of behavioral changes for prevention and control. Therefore, there is an urgent need to leverage the gains made in the management of HIV/AIDS by integrating NCDs care into HIV care. Integration of NCDs care into HIV has successfully been done in Western Kenya through the Academic Model Providing Access to Healthcare (AMPATH) model of care, an academic medical partnership between North American and Kenyan partners based in western Kenya town of Eldoret. Such an approach is recommended in other HIV care models.

## 5. Conclusion

The prevalence of hypertension is high among HIV patients. Traditional cardiovascular risk factors were associated with hypertension, but no association was observed with ART regime or duration of ARV use. There is a need to integrate hypertension management into the regular HIV care at Thika level 5 Hospital.

## Figures and Tables

**Table 1:** Sociodemographic and clinical characteristics of the study sample.

Variables	Male *n* = 293 Mean (SD) or % (*n*)	Female *n* = 646 Mean (SD) or % (*n*)	Total *n* = 939 Mean (SD) or % (*n*)
Age in years	47 (11.5)	43 (10.2)	44 (10.7)
SBP (mmHg)	127.9 (17.9)	121.3 (17.8)	123.4 (18.1)
DBP (mmHg)	75.5 (11.7)	73.4 (10.1)	74.1 (10.7)
Weight (kg)	66.5 (13.1)	65.4 (13.5)	65.7 (13.4)
Height (meters)	1.70 (0.1)	1.59 (0.1)	1.62 (0.1)
BMI (kg/m^2^)	23.1 (4.2)	25.8 (5.1)	24.9 (4.9)

*BMI categories*			
Underweight (BMI ≤18.4)	9.9 (29)	56.7 (38)	7.1 (67)
Normal (BMI 18.5–24.9)	61.8 (181)	40.4 (261)	47.1 (442)
Overweight/obese (BMI≥25)	28.3 (83)	53.7 (347)	45.8 (430)

*Blood pressure categories*			
Normal BP (SBP < 130 and DBP < 85 mmHg)	59.7 (175)	70.6 (456)	67.2 (631)
Prehypertensive (SBP of 130–139 and/or DBP 85–89 mmHg)	15.0 (44)	13.5 (87)	131 (13.9)
Hypertensive (SBP ≥140 and/or DBP ≥ 90 mmHg)	25.3 (74)	15.9 (103)	18.9 (177)

*ARV drugs*			
TDF/3TC/EFV	12 (35)	40.6 (262)	31.6 (297)
D4T/3TC/NVP	15.4 (45)	16.7 (108)	16.3 (153)
TDF/3TC/NVP	1.7 (5)	7.6 (49)	5.8 (54)
TDF/3TC/DTG	50.5 (148)	14.9 (96)	26 (244)
TDF or AZT/3TC/LPV/*r* or ATV/*r*	4.8 (14)	3.9 (25)	4.2 (39)
AZT/3TC/NVP	3.1 (9)	7.7 (50)	6.3 (59)
3TC/D4T/EFV	8.9 (26)	5.4 (35)	6.5 (61)
Others	3.8 (11)	3.2 (21)	3.4 (32)

*ART regimen*			
NNRTIs and NRTIs	38.6 (113)	77.6 (501)	65.4 (614)
NRTIs and PIs	5.8 (17)	4.6 (30)	5.0 (47)
NRTIs and INSTIs	55.6 (163)	17.8 (115)	29.6 (278)

*Duration of ARV therapy*			
<2 years	13.3 (39)	10.8 (70)	11.6 (109)
≥2 years	86.7 (254)	89.2 (576)	88.4 (830)

*History of smoking*			
Yes	14.3 (42)	1.9 (12)	5.8(54)
No	85.7 (251)	98.1 (634)	94.2(885)

*History of alcohol consumption*			
Yes	22.1 (59)	6.1 (39)	11.1 (95)
No	77.9 (208)	93.9 (550)	88.9 (758)

SBP: systolic blood pressure; DBP: diastolic blood pressure; BMI: body mass index; ARV: antiretroviral; ART: antiretroviral therapy; AZT: zidovudine; 3TC: lamivudine; NVP: nevirapine; EFV: efavirenz; TDF: tenofovir disoproxil fumarate; DTG: dolutegravir; D4T: stavudine; LPV/*r*: lopinavir/ritonavir; ATV/*r*: atazanavir/ritonavir; NNRTIs: nonnucleoside reverse transcriptase inhibitors; NRTI: nucleoside and nucleotide reverse transcriptase inhibitors; INSTIs: integrase strand transfer inhibitor.

**Table 2:** Bivariate associations of patients' characteristics versus hypertension among HIV patients.

Characteristic	Hypertensive *n* = 177% (*n*)	Normotensive *n* = 762% (*n*)	*χ*2 *p* value
*Sex*			
Female	15.9 (103)	84.1 (543)	0.001
Male	25.2 (74)	74.7 (219)	

*Age*			
31–40	6.9 (23)	93.1 (310)	<0.001
41–50	20.4 (74)	79.6 (288)	
51–60	32.6 (58)	67.4 (120)	
>60	33.3 (22)	66.7 (44)	

*Marital status*			
Divorced/separated	15.4 (24)	84.6 (132)	0.03
Married	21.1 (102)	78.9 (383)	
Single	13.1 (25)	86.9 (166)	
Widowed/widower	24.3 (25)	75.7 (78)	

*BMI categories*			
Underweight (BMI ≤18.4)	4.5 (3)	95.5 (64)	<0.001
Normal (BMI 18.5–24.9)	14.2 (63)	85.8 (379)	
Overweight/obese (BMI ≥ 25)	25.8 (111)	74.2 (319)	

*History of smoking*			
Yes	7.9 (14)	5.3 (40)	0.5
No	92.1 (163)	94.7 (722)	

*History of alcohol consumption*			
Yes	20.9 (34)	8.8 (61)	<0.001
No	79.1 (129)	91.2 (629)	

*ART regimen*			
NNRTIs and NRTIs	16.5 (108)	83.5 (548)	0.02
NRTIs and PIs	25.6 (10)	74.4 (29)	
NRTIs and INSTIs	24.2 (59)	75.8 (185)	

*Duration of ARV therapy*			
<2 years	16.5 (18)	83.5 (91)	0.5
≥2 years	119.2 (159)	80.8 (671)	

**Table 3:** Adjusted multivariate logistic regression model for factors associated with hypertension among HIV patients.

	Hypertension			
Variables	AOR	SE	95% CI	*p* value
*Sex*				
Female	Ref			
Male	2.02	0.48	1.26–3.24	0.003

*Age*				
18–40	Ref			
41–50	2.80	0.77	1.63–4.81	<0.001
51–60	4.74	1.43	2.62–8.56	<0.001
>60	6.31	2.44	2.96–13.47	<0.001

*Marital status*				
Divorced/separated	Ref			
Married	1.11	0.30	0.63–1.93	0.72
Single	1.01	0.35	0.51–2.00	0.96
Widowed/widower	1.72	0.61	0.86–3.47	0.13

*BMI categories*				
Normal (BMI 19–25)	Ref			
Underweight (BMI ≤18)	0.31	0.20	0.09–1.10	0.07
Overweight (BMI 26–29)	2.14	0.44	1.43–3.20	<0.001
Obese (BMI ≥30)	2.77	0.69	1.70–4.51	<0.001

*History of smoking*				
No	Ref			
Yes	0.61	0.25	0.27–1.38	0.23

*History of alcohol consumption*				
No	Ref			
Yes	2.68	0.81	1.49–4.84	<0.001

*ART regimen*				
NNRTIs and NRTIs	Ref			
NRTIs and PIs	1.40	0.64	0.56–3.46	0.49
NRTIs and INSTIs	0.83	0.19	0.53–1.31	0.45

*Duration of ARV therapy*				
<2 years	Ref			
≥2 years	1.10	0.38	0.55–2.20	0.77

## Data Availability

Data from this study are available from the approving ethics committee (Moi University College of Health Sciences/Moi Teaching and Referral Hospital Institutional Research and Ethics Committee) upon written requests under the ethical provisions of the body. To access data, one can write to Board Chair, Institutional Research Ethics Committee (IREC), Moi University College of Health Sciences and Moi Teaching and Referral Hospital, P.O. Box. 3–30100 Eldoret, Kenya (Office line: +254787723677; e-mail: irecmtrh@gmail.com or contact@irec.or.ke).
